# Synergistic regulation mechanism of iperoxo and LY2119620 for muscarinic acetylcholine M2 receptor†

**DOI:** 10.1039/c8ra01545g

**Published:** 2018-04-09

**Authors:** Quan Li, Hai-Feng Chen

**Affiliations:** State Key Laboratory of Microbial Metabolism, Department of Bioinformatics and Biostatistics, National Experimental Teaching Center for Life Sciences and Biotechnology, College of Life Sciences and Biotechnology, Shanghai Jiaotong University Shanghai 200240 China haifengchen@sjtu.edu.cn +86-21-34204348 +86-21-34204348; Shanghai Center for Bioinformation Technology Shanghai 200235 China

## Abstract

Muscarinic acetylcholine receptors are GPCRs that regulate the activity of a diverse array of central and peripheral functions in the human body, including the parasympathetic actions of acetylcholine. The M2 muscarinic receptor subtype plays a key role in modulating cardiac function and many important central processes. The orthosteric agonist and allosteric modulator can bind the pocket of M2. However, the detailed relationship between orthosteric agonist and allosteric modulator of M2 is still unclear. In this study, we intend to elucidate the residue-level regulation mechanism and pathway *via* a combined approach of dynamical correlation network and molecular dynamics simulation. Specifically computational residue-level fluctuation correlation data was analyzed to reveal detailed dynamics signatures in the regulation process. A hypothesis of “synergistic regulation” is proposed to reveal the cooperation affection between the orthosteric agonist and allosteric modulator, which is subsequently validated by perturbation and mutation analyses. Two possible synergistic regulation pathways of 2CU-I178-Y403-W400-F396-L114-Y440-Nb9 and IXO-V111-F396-L114-Y440-Nb9 were identified by the shortest path algorithm and were confirmed by the mutation of junction node. Furthermore, the efficiency of information transfer of bound M2 is significant higher than any single binding system. Our study shows that targeting the synergistic regulation pathways may better regulate the calcium channel of M2. The knowledge gained in this study may help develop drugs for diseases of the central nervous system and metabolic disorders.

## Abstract 2

Dynamics correlation network was used to reveal the synergistic regulation mechanism of iperoxo and LY2119620 for muscarinic acetylcholine M2 receptor.

## Graphical abstract

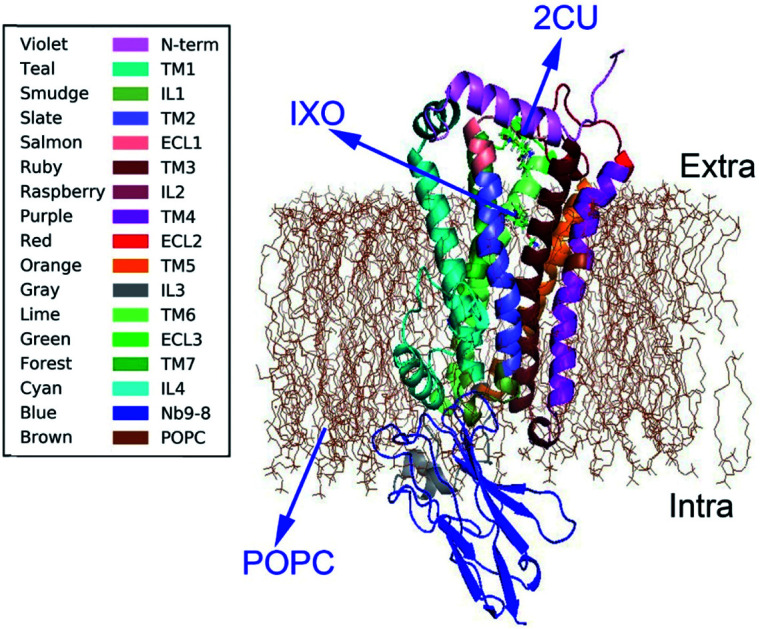

## Introduction

Muscarinic receptors are involved in a large number of physiological functions including regulation of heart rate and contractile forces, contraction of smooth muscles, and the release of neurotransmitters.^1^ There are five subtypes of muscarinic AChRs based on pharmacological activity: M1–M5 which regulate the activity of a diverse array of central and peripheral functions in the human body, including the parasympathetic actions of acetylcholine.^2^ All five are found in the CNS, while M1–M4 are also found in various tissues.^3^ The activation of the M2 receptor in the heart is important for closing calcium channels in order to reduce the force and rate of heart contraction.^4^ Hormonal, neurotransmitter, visual, and olfactory signaling is largely regulated by a versatile class of membrane receptors, referred to as G-protein coupled receptors (GPCRs).^5^ The family of the muscarinic acetylcholine receptors has been widely studied as a model system for the interaction of allosteric modulators with GPCRs.^6^ These GPCRs become important targets for agonist and antagonist drug design.

**Fig. 1:**
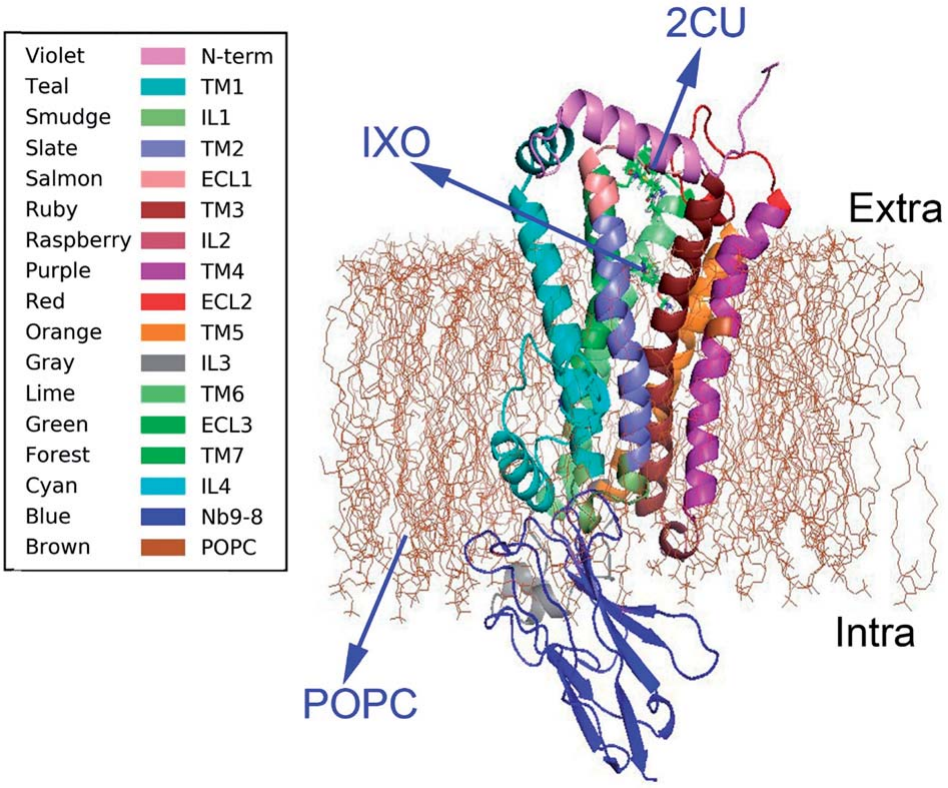
The structure of M2 and membrane. Violet color represents N terminal domain, teal for TM1, smudge for IL1, slate for TM2, salmon for ECL1, ruby for TM3, raspberry for IL2, purple for TM4, red for ECL2, orange for TM5, gray for IL3, lime for TM6, green for ECL3, forest for TM7, cyan for IL4, blue for Nb9-8, brown for POPC membrane.

Muscarinic receptors have attracted particular interest due to their ability to bind small molecule allosteric modulators.^7^ Many crystal structures were recently obtained for active states of the M2 and M3 muscarinic receptors,^8,9^ including structures of a GPCR bound to an allosteric modulator. The crystal structure of M2 muscarinic receptor was released in 2013 (pdb code: 4MQS).^2^ The structure (Fig. 1) with POPC membrane includes an extracellular amino terminus, seven *trans*-membrane helices, a cytoplasmic carboxyl terminus, extracellular and intracellular loops, and two binding sites for orthosteric agonist iperoxo (IXO) and positive allosteric modulator of LY2119620 (2CU). The agonist IXO can enhance the effect of allosteric modulator 2CU.^10^ However, the relationship between orthosteric agonist IXO and allosteric modulator 2CU is still unclear, as well as how the agonist and allostery information transfer the regulation information from binding site to G-protein interaction area. In this study we intend to answer the following questions. (1) Is there a synergistic regulation mechanism? (2) How does the information transfer from the regulation sites to the binding site of Nb9-8? And (3) what are the regulation pathways in M2?

To answer these questions, dynamics correlation networks were constructed based on all-atom molecular dynamics (MD) simulations of the wild type and mutant M2 receptor. From the comparisons of the networks between bound and free M2, a hypothesis of “synergistic regulation” and two possible synergistic regulation pathways were proposed to explain the agonist enhancing the allosteric effect, which are subsequently validated by both network weaken and mutation analyses.

## Materials and method

### Molecular dynamics simulation

The atomic coordinates of the active M2 in complex with Nb9-8 and IXO are extracted from the Protein Data Bank (pdb code: 4MQS), the coordinates of bound M2 from 4MQT, free M2 from 4MQS, and M2/2CU from 4MQT removing ligand.^2^ D103E and Y403A are the mutants mentioned in the previous works to reveal the activation mechanism of IXO and 2CU. The mutant structures were constructed using SYBYL-X 2.1.1.^11^ All structural visualizations were conducted in PyMOL 1.7.9.^12^

All initial structures were first minimized in SYBYL-X 2.1.1 to eliminate any possible overlaps or clashes. As the M2 receptor is a membrane protein, a protein/membrane complex was generated by CHARMM-GUI web-based Membrane Builder, M2 protein was loaded in POPC membrane before MD simulation. AMBER12 was used to perform MD simulations with periodic boundary conditions.^13^ Hydrogen atoms were added using the LEaP module of AMBER12. Counterions were used to maintain system neutrality. All systems were solvated in a truncated octahedron box of TIP3P waters with a buffer of 10 Å. The pairwise interactions (van der Waals and direct Coulomb) were computed with a cutoff distance of 8 Å. Particle mesh Ewald (PME) was employed to treat long-range electrostatic interactions in AMBER12.^14^ The improved ff99SBildn force field was used for the intramolecular interactions. The Langevin thermostat was used in the preparation runs with a friction constant of 1 ps^−1^ and the Berendsen thermostat was used in the production runs.^12,13,15^ All MD simulations were accelerated with the CUDA version of PMEMD in GPU cores of NVIDIA Tesla K80.

To relieve any further structural clash in the solvated systems, initial minimization with macromolecule frozen was performed using 500-step steepest descent minimization and 2000-step conjugate gradient minimization. Next the whole system was followed by 1000-step steepest descent minimization and 19 000-step conjugate gradient minimization. After minimization, a 400 ps heating up and a 200 ps equilibration in the NVT ensemble at 310 K were performed before MD simulation was conducted in the NPT ensemble at 310 K. To compare the difference among the chosen M2 systems, seven systems were simulated and the detailed simulation conditions are listed in Table 1.

**Table 1:** Simulation conditions for seven systems

System	Composition	Temp. (K)	Time (ns)	Traj.	Ions
Free M2	M2	310	160	5	
M2/2CU	M2/2CU			5	
M2/IXO	M2/IXO			5	
Bound M2	M2/IXO/2CU			5	Na^+^
Y403A	M2/IXO/2CU			1	
F396A	M2/IXO/2CU			1	
D103E	M2/IXO			1	

### Interaction analysis

Interaction analysis was handled with in-house software.^16,17^ Residues, substrates, or effectors are in hydrophobic interaction when mass centers of their side chains or ligands are closer than 6.5 Å. A previous study has shown that residue charge–charge interactions up to 11 Å were found to contribute to protein/protein binding free energies.^18,19^ Thus, electrostatic (*i.e.*, charge–charge) interactions are assigned when the distances between mass centers of their side chains are less than 11 Å. Hydrogen bond is defined that the distance between the donor and acceptor is less than 3.5 Å and the bond angle is larger than 120°.^17,20–22^

### MM/PBSA free energy calculation

Free-energy calculation together with MD simulations can provide qualitative predictions of M2–ligand binding energies. The free energy of binding (Δ​*G*_bind_) is estimated by eqn (1).

1 δ*G*_bind_ = *G*_R+L_ − (*G*_R_ + *G*_L_)

where *G*_R+L_, *G*_R_, and *G*_L_ is the free energies of the complex, the isolated M2 and ligand, respectively. In the MM/PBSA approach,^20,23–25^ each free energy term in eqn (1) is calculated as eqn (2).

2 *G* = *E*_bond_ + *E*_vdw_ + *E*_elec_ + *G*_PB_ + *G*_SA_ − TS

where *E*_bond_ is the molecular mechanics bond energy, which is the sum of bond, angle and dihedral energies; *E*_vdw_ is the molecular mechanics van der Waals energy contribution; *E*_elec_ is the molecular mechanics electrostatic energy; *G*_PB_ and *G*_SA_ are polar and non-polar contributions to the solvation energy; *T* is the absolute temperature and *S* is the solute entropy. All the binding energies were calculated using the PBSA model in MMPBSA.^18,23–25^ Considering that the equilibration period may affect the result, only the last 50 ns of MD trajectories was used to calculate the binding free energy taking snapshots every 50 ps.

### Correlation network analysis

Every amino acid or ligand was defined as one node for dynamics network.^26^ The fluctuation correlation between any pair of nodes was calculated with eqn (3).3
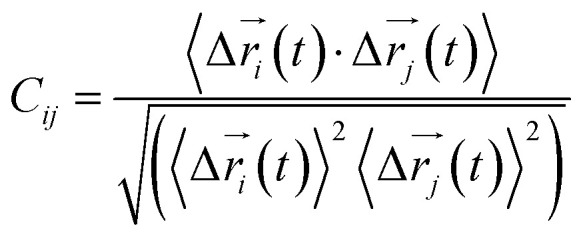
where 
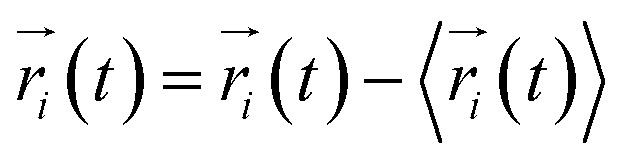
, 
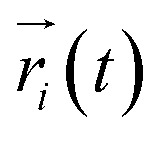
 is the position of node *i* at time *t*, and 〈·〉 represents time averaging, and *r*_*i*_(*t*) is the position of node *i* at time *t*. These elements were conveniently organized as a covariance matrix for a simulated system. In the current study, the covariance matrix for each system was constructed using snapshots (every 2 ps) in the last 50 ns of all simulated trajectories. Besides nodes, “edge” that transfers regulation information from one node to another is also a key concept in network construction. An edge is defined between any two nodes without covalent bond but with heavy atoms closer than 4.5 Å over 75% sampling time. The strength of the edge is defined as the absolute value of the inter-node correlation (*C*_*ij*_) between nodes *i* and *j*. The number of connected edges at each node is defined as the degree of the node. Correlation-weighted degree, which is the summation of strengths of all edges connected to a given node, indicates the importance of the node. After the network construction, network topological analyses were performed using Cytoscape3.1.1.^27^ For community analyses, the Girvan–Newman algorithm was utilized. Most of the network analysis tools utilized here were developed by the Luthey-Schulten Group^27–29^ and the strategy of process was successfully used in the allostery of riboswitch and protein.^30–38^

### Shortest-path length (SPL) analysis

The Dijkstra algorithm was used to calculate the shortest path between any two nodes in the network.^39^ The SPL is defined as the distance between all pairs of nodes in the network. The length of shortest path between two nodes is the shortest travelling distance from one node to another node along the network edges. This method has been used previously to predict residues important in allosteric signaling within protein families.^40^

## Results

### Stability of M2 in free and bound states

Cα RMSD relative to the initial structure shows that 160 ns simulations are sufficient for the equilibration of the wild types and mutant systems at 310 K (ESI Fig. S1†). Root mean squared fluctuations of Cα atom for four systems are shown in ESI Fig. S2.† Cα variation of bound M2 in the regions of 180–200 and 320–380 are much higher than other three systems, this indicates that there is significant conformation change in these areas upon the binding of IXO and 2CU. These areas locate at extracellular loop2 (ECL2) and junction linking TM7 and Nb9-8. In order to evaluate the conformational adjustment of bound M2, the pairwise Cα distance difference between bound and free M2 was analyzed. The conformational adjustment of M2 upon binding IXO and 2CU was shown in ESI Fig. S3.† This figure shows that the visible conformation change is located at the regions of ECL2, TM6, IL4, and Nb9-8 which are consistent with the RMSF results. The deep color represents M2 structure extending at ECL2 and IL4 regions. For TM6 and Nb9-8 regions, complex conformation changes were found and might construct a signaling pathway.

**Fig. 2:**
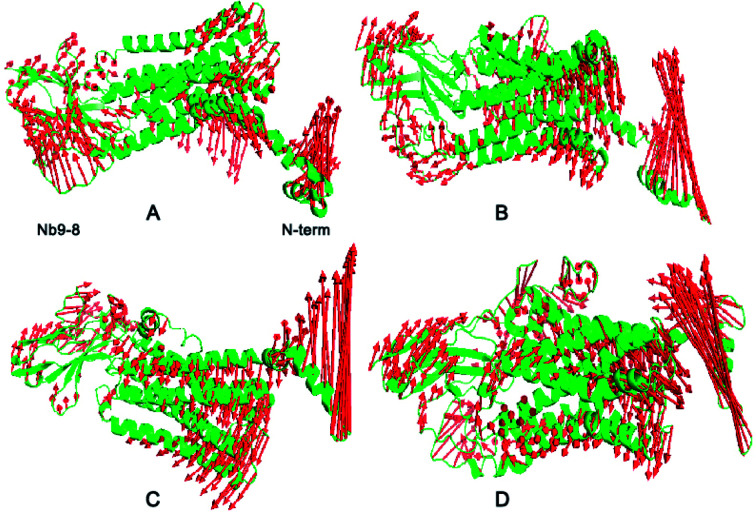
PCA analysis for four systems. (A) Free M2. (B) M2/2CU. (C) M2/IXO. (D) Bound M2. The left polar represents Nb9-8 area, the right polar for the first 20 residues, and the middle part close to the right for the binding site of IXO and 2CU.

To quantitatively identify the conformational adjustment, the principal component analysis (PCA) was carried out on the four systems to study the motion. The first three most dominant components (named PC1, PC2, and PC3) represent over 95% of the overall fluctuations in each system (ESI Fig. S4†), it showed the Ecl2, TM5-TM7 and Nb9-8 areas contribute most of the fluctuations with shearing motion. To clearly display the motion, the results of each system are shown in Fig. 2. For the free M2, some conformational changes were observed. Upon binding allosteric effectors of IXO and 2CU, N terminal domain of bound M2 are in the opening motion mode. This motion was induced by the binding of allosteric effectors. We can also find similar motion mode for N terminal domain of M2/2CU and M2/IXO.

To confirm the reliability and robustness of MD simulations for membrane protein M2, the binding free energies between allosteric effector (2CU or IXO) and M2 for the wild type and mutants are calculated with MMPBSA^18,21,23,24^ and listed in Table 2. Although there is limitations in MMPBSA approach, it is still possible to discover some interesting conclusions from the different systems.^41^ The MMPBSA analysis shows that the binding energy between M2 and IXO is −41.87 kcal mol^−1^, it is always much lower than that of mutant D103E with −37.73 kcal mol^−1^. This is in qualitative agreement with experiment in that the D103E mutant significantly decreases the binding affinity of IXO.^2^ At the same time, the binding free energy between IXO and M2 for the Y403A mutant is about 3.82 kcal mol^−1^ higher than that of bound M2. This is also consistent with experiment in that Y403A mutant resulted in significantly reduced affinity for the orthosteric agonists.^42^ In summary, the MD simulation and binding free energy analysis are in good accord with previous experiment, indicating the good quality of the current model of M2.

**Table 2:** Binding free energies (kcal mol^−1^) between allosteric effectors and M2

System	Δ*G* between receptor and IXO
M2/IXO	−41.87 ± 3.21
D103E	−37.73 ± 2.61
Bound M2	−43.54 ± 1.90
Y403A	−38.05 ± 3.15
F396A	−28.40 ± 2.11

### Correlation networks of free and M2 complex are different

To reveal the allosteric mechanism, the dynamic correlation network analysis based on the covariance matrices calculated by in-house software was performed to illustrate the residue fluctuation correlation network and the network parameters for each system are listed in Table 3, the map of network is shown in ESI Fig. S5.†

**Table 3:** Network parameters for free and bound M2 systems

Parameters	Free M2	M2/2CU	M2/IXO	Bound M2
Number of nodes	476	477	477	478
Max degree	15	16	15	15
Avg. degree	7.67	7.58	7.63	7.86
Nodes numbers of degree > 10	47	51	46	61
Max edge betweenness	0.080	0.090	0.070	0.11
Max node betweenness	0.18	0.27	0.19	0.31
Edge number	1846	1851	1829	1886

Table 3 indicates that the values of network parameter for bound M2 are the highest among these four systems for max average degree, max edge betweenness, the node betweenness, and the nodes number of degree > 10. This suggests that the network characters for bound M2 are significant different from those of other systems Y403, W400, F396, L114, V129, and F274 with high degree were marked in the bound M2 network and most of them were found on the shortest-pathway to be discussed below. This indicates that the allosteric effectors indeed increase the correlation of nodes for bound M2.

**Fig. 3:**
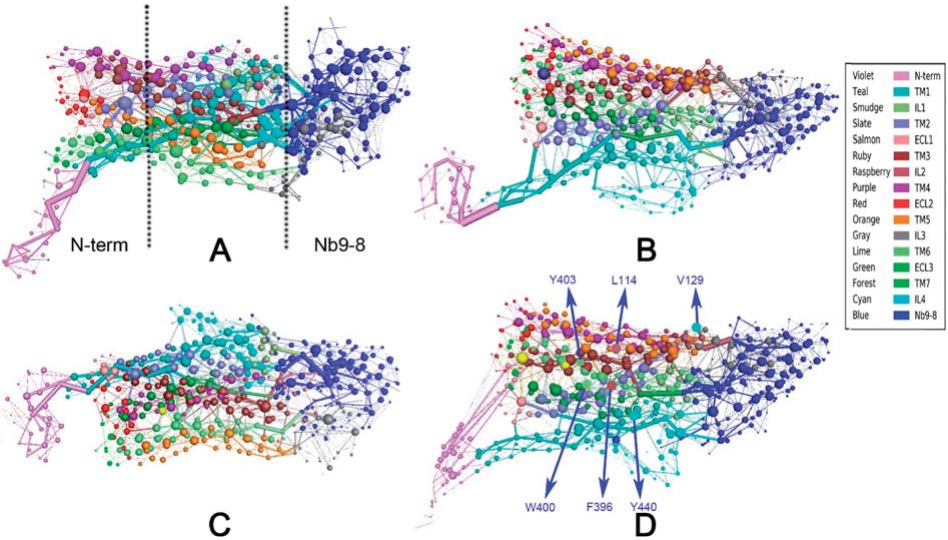
2D-projection of correlation network for each system. (A) Free M2. (B) M2/2CU. (C) M2/IXO. (D) Bound M2. The protein was segmented for 16 domains represented by different colors, the blue nodes represent the Nb9-8 area, the binding area locates in extracellular and the IXO and 2CU are colored yellow in bound M2.

To measure the distribution of weighted nodes with high degree and betweenness in each system, the distribution map is shown in ESI Fig. S6.† This figure indicates that the nodes of bound M2 with weighted degree larger than 10 are focused on Nb9-8 and transmembrane regions. The nodes of bound M2 with weighted betweenness are mostly located at the segments of TM3, TM6, TM7, and Nb9-8. These results are shown that the bound M2 has a significant different information transfer network from other systems. The 2D-projection of correlation networks is shown in Fig. 3 for free M2, M2/IXO, M2/2CU, and bound M2 which reveal the whole network with nodes and edges.

### Community networks are different

**Fig. 4:**
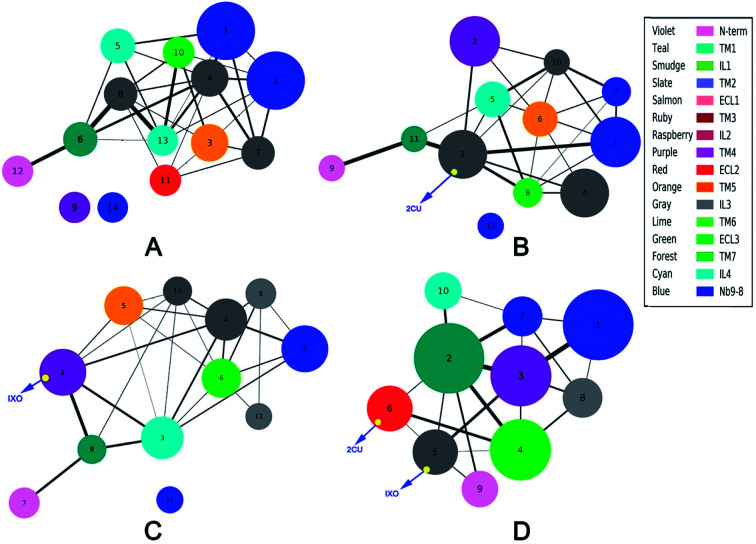
Community network for each system. (A) Free M2. (B) M2/2CU. (C) M2/IXO. (D) Bound M2. The domains are regrouped in communities based on Girvan–Newman algorithm and the color represents the domain.

The analysis of correlation network shows that the networks are significantly different among the four systems. To reveal the information transfer pathways, the Girvan–Newman was used to split these networks into communities. The community of four systems are shown in Fig. 4. This figure indicates that the community network of the free M2 has 14 communities including two isolated clusters, however the community network becomes more centralized upon binding to 2CU and/or IXO. There are only 12 communities with one isolated cluster for M2/2CU; 12 communities with one isolated cluster for M2/IXO, and 10 communities without isolated cluster for the bound M2. This demonstrates that the effects of 2CU and IXO binding can optimize the integrity of the community in the complexes.^2^ The information flow can freely transfer from 2CU and IXO regulation sites to the binding site of Nb9-8.

Furthermore, structural analysis indicates that there is one hydrogen bond, five hydrophobic, and seven electrostatic interactions between 2CU and M2, and one hydrogen bond and six hydrophobic interactions between IXO and M2 with population higher than 40% (ESI Fig. S7†) in the bound M2. Thus orthosteric agonist IXO and allosteric modulator 2CU introduce strong interactions with M2. Furthermore, the binding free energy between 2CU and M2 for bound M2 is significantly lower than that for M2/2CU (listed in Table 4). Therefore, the hypothesis of “synergistic regulation mechanism” was proposed to explain the regulation mode for M2. The previous work reports that 2CU shows strong positive cooperation with iperoxo and selectively enhances the affinity of the orthosteric agonist for the M2.^2^

**Table 4:** Binding free energies (kcal mol^−1^) between M2 and 2CU or IXO

System	Δ*G* between receptor and 2CU	System	Δ*G* between receptor and IXO
M2/2CU	−32.29 ± 2.64	M2/IXO	−41.87 ± 3.21
Bound M2	−34.74 ± 2.01	Bound M2	−43.54 ± 1.90

### Modifications to perturb the community networks

In order to validate the hypothesis of synergistic regulation, modifications to weaken the interactions between IXO or 2CU and M2 for bound M2 were used to study the effects on the community networks. These modifications were realized by just deleting the edges between 2CU or IXO and M2 nodes in the network. The community networks of weakened systems are shown in ESI Fig. S8.† Weakening operations will induce more fragmented community networks and decrease the efficiency of information transfer. For weakened IXO, the number of community increases from 10 to 15 including two isolated vertexes and the Nb9-8 community becomes fragmented. For weakened 2CU, the number of community number increases from 10 to 17 with two isolated nodes and the information could not transfer from 2CU to the effector site of Nb9-8. When weakening both IXO and 2CU, the community number increases from 10 to 17 with 4 isolated nodes. Furthermore, Nb9-8 domain are split into three communities and more than that of bound M2. In summary, these modifications lead to significant repartition of the community networks. This finding confirms that the interactions between 2CU/IXO and M2 indeed influence the community network and further support our hypothesis of synergistic regulation mechanism.

### Validation of regulation mechanism by mutation

**Fig. 5:**
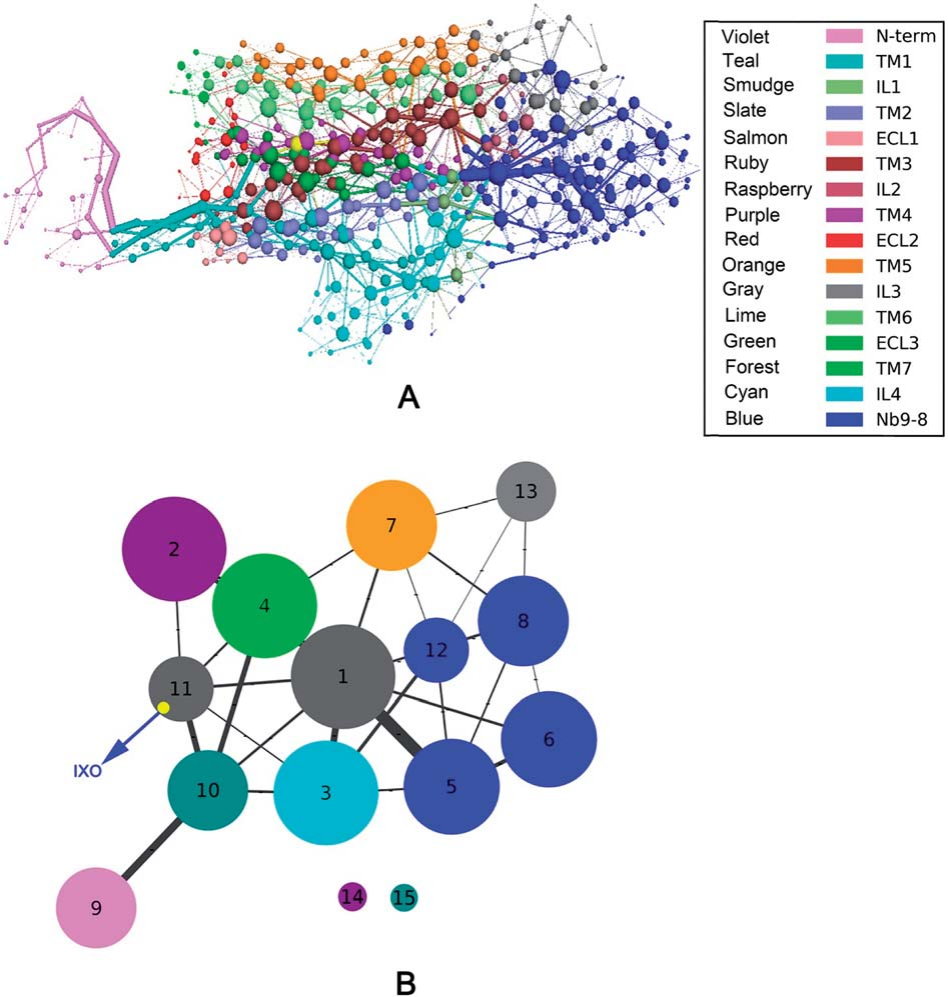
Correlation network and community network for D103E mutation. (A) Correlation network. (B) Community network.

In order to evaluate the receptor activation of M2 by IXO, the D103E mutation of M2 was analyzed in a previous experiment,^2^ demonstrating that D103E abolished agonist-induced M2 receptor activation and reduced 380-fold affinities for iperoxo. To further evaluate the hypothesis of “synergistic regulation mechanism”, the network of D103E is constructed and shown in Fig. 5A, indicating that the network is different from that of wild type for bound M2. The community network was shown in Fig. 5B. This figure shows that the number of community increase from 12 to 15 with two isolated clusters in D103E compared with the wild type. This suggests that D103 plays a key role in information transfer of bound M2, this is consistent with the previous works that D103E resulted in significantly reduced affinity for the orthosteric agonist. The interaction between IXO and M2 was shown in ESI Fig. S9† which is significant reduced. In summary, even if the information can transfer from the IXO orthosteric site to the effector site of Nb9-8, the efficiency is reduced due to mutant of D103E. These results further confirmed the hypothesis of synergistic regulation mechanism.

### Synergistic regulation pathway for M2 complex

The network and community analyses of the wild type and mutants confirm that the IXO and 2CU binding induces synergistic activation of M2. Next, it is natural to identify the synergistic regulation pathway based on the shortest path analysis which was used to identify the regulation pathway between the regulation sites and the binding site of Nb9-8. All he shortest-pathway length (SPL) was calculated from allosteric site to effector site of Nb9-8, the possible pathways with varies SPL were shown in ESI Fig. S10.† It indicates the information flow can transfer from the binding site to effector site *via* different pathways. The average shortest-pathway length (SPL) for each system is listed in Table 5.

**Table 5:** The SPL in M2 complex

System	Shortest pathway	Nodes numbers	SPL
M2/2CU	2CU-A191-W155-A194-V111-L115-F119-C124-K221-E223-Nb9	9	6.60 ± 1.00
M2/IXO	IXO-S151-W400-N436-Y440-R121-Nb9	5	4.40 ± 1.10
F396A	2CU-W422-G425-L428-I431-T434-T37-G40-L43-F451-Nb9	9	5.63 ± 1.10
	IXO-W400-Y196-V199-T203-W207-A212-R216-K218-E220-Nb9	9	7.58 ± 1.20
Y403A	2CU-Y104-A109-W148-I144-S64-Y60-N58-N444-Nb9	8	5.89 ± 1.10
	IXO-N108-N113-S64-Y60-N58-N444-Nb9	6	7.96 ± 1.20
Bound M2	2CU-I178-Y403-W400-F396-L114-Y440-Nb9	6	4.20 ± 0.90
	IXO-V111-F396-L114-Y440-Nb9	4	3.60 ± 0.80

**Fig. 6:**
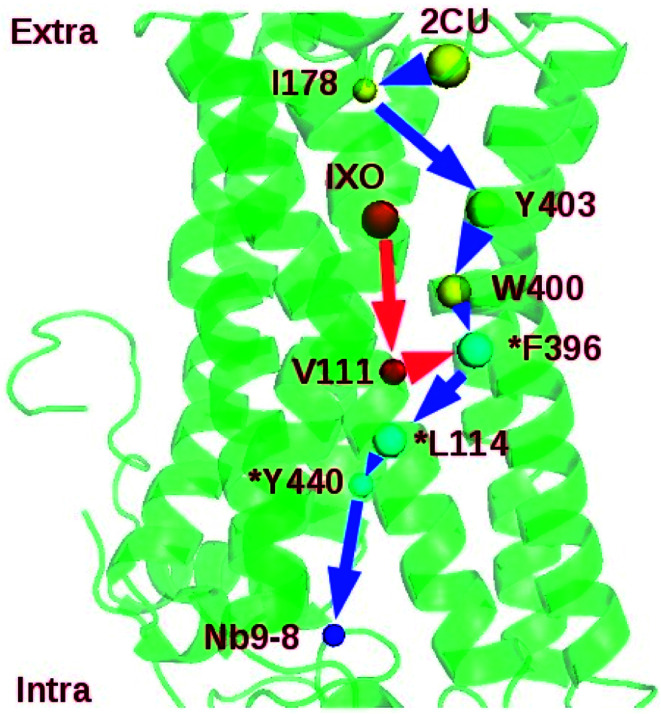
Shortest synergistic regulation pathway of bound M2.

Two regulation pathways were identified in the bound M2: 2CU-I178-Y403-W400-F396-L114-Y440-Nb9 and IXO-V111-F396-L114-Y440-Nb9 (Fig. 6). This indicates that I178, Y403, W400, F396, L114, Y440, and V111 are key nodes for information transfer in bound M2. These strongly correlated residues may play critical roles in transferring the information of regulation. Note also that the SPL for the community network of bound M2 (7.80) is also shorter than the sum of SPLs of similar pathways in M2/IXO (4.40) and M2/2CU (6.60). This indicates that the efficiency of information transfer is 42.55% for M2/IXO and 27.07% for M2/2CU if the bound M2 efficiency is 100%. This further supports the hypothesis of synergistic regulation mechanism.

Mutation and network perturbation were used to validate the two regulation pathways. Y403 is one of the key residues on the shortest pathway from 2CU to Nb9. The dynamics correlation network of Y403A is shown in ESI Fig. S11.† This indicates that the network is significant different from that of wild type. The SPL of mutant Y403A was also listed in Table 5, indicating that the SPL for Y403A was much longer than that for wild type. The efficiency of information transfer for Y403A is just 56.41% of wild type. This suggests that Y403 plays a key role in the information transfer and confirms the regulation pathway. This is consistent with the previous work that Y403A decreased the efficacy and functional affinity of agonist Ach.^42^ At the same time, F396 is at the junction of the two proposed pathways and might be crucial for the information flow. In order to further confirm the key function of F396, mutant F396A was simulated for 160 ns. The binding free energy between IXO and M2 was about −28.40 kcal mol^−1^ and much higher than that of wild type. The community of F396A is shown in ESI Fig. S12† with one isolated cluster. The number of community increases from 10 of bound M2 to 15. Therefore, the efficiency of information flow is significantly reduced to 59.05% due to the fragmentation of the network. This suggests that the proposed regulation pathways, at least F396 before the junction, play key roles in the synergistic regulation. The previous works also confirm that the mutations of Y403, W400, and F396 in M2 decrease the binding affinity of agonist or efficacy.

## Discussion

Previous work reports that 2CU can directly activate the M2 receptor and enhance the affinity of IXO.^2^ Furthermore, 2CU has strong positive cooperative with IXO and enhances the potency of agonists.^43^ D103E mutant experiment reduced the decrease of the binding affinities for IXO (∼380-fold).^2^ In simulation, the binding free energy increased about 1.67 kcal mol^−1^ for D103E mutant, consistent with the previous work.^2^

**Fig. 7:**
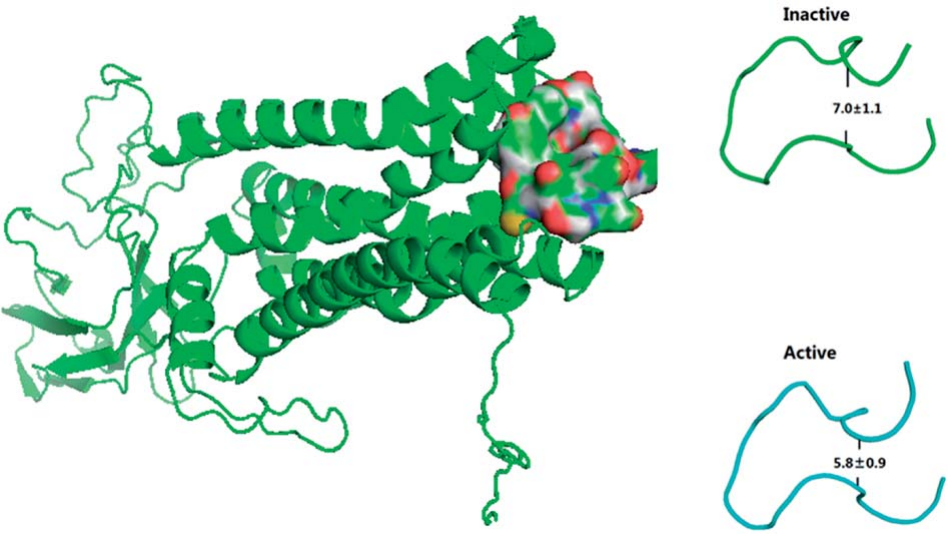
The ECL2 mouth is closed in the active state.

The previous work reports that ECL2 mouth becomes smaller upon the activation of M2 by IXO or 2CU to prevent other ligand from moving into the closed, active extracellular vestibule.^8^ TM5 and TM6 are closer to each other in M2-active state than those in the inactive state.^2^ The conformer of ECL2 mouth from MD simulation is shown in Fig. 7, which indicates that the dimension of ECL2 mouth decreases from 7.0 Å to 5.8 Å upon the binding of IXO and 2CU. The close propensity could prevent other ligand to enter into extracellular vestibule, this is consistent with the previous work. The alignment between inactive and active states for bound M2 is shown in ESI Fig. S13,† it indicates that TM5 and TM6 are closer in the active state than in the inactive state. The previous work reports that 2CU forms two hydrogen bonds with TRP422, and ASN410 and induces conformation change in binding area of ext-TM4-TM5-TM6.^2^ The hydrogen bond interaction between 2CU and M2 is shown in ESI Fig. S14.† This figure shows that 2CU has two hydrogen bonds with these residues to form a hydrogen bond network. These results are in agreement with the previous work.

## Conclusion

Residue/residue fluctuation correlation network and shortest-pathway analysis were used to reveal the regulation mechanism of M2 upon binding to allosteric effectors of 2CU and agonist IXO. The results suggest that the dynamics correlation network of bound M2 has more hub nodes than that of free M2, M2/2CU, or M2/IXO. The community network of the bound M2 is clustered into a complete community without isolated cluster. However, there are two isolated clusters for free M2. The information flow can freely transfer from the IOX and 2CU regulator sites to the binding site of Nb9-8 for bound M2. The efficiency of information transfer for bound M2 is higher than that of M2/2CU or M2/IXO. The binding free energy between the antagonist IXO and M2 for the bound M2 is significantly lower than that of M2/2CU or M2/IXO system. Therefore, we propose the hypothesis of synergistic regulation in bound M2 system and the hypothesis was validated through mutation and network perturbation. At the same time, two regulation pathways were found and confirmed by Y403A and F396A mutants.

## Conflicts of interest

The authors declare that there is no conflict of interest.

## Supplementary Material

RA-008-C8RA01545G-s001

## References

[cit1] Abrams P., Andersson K. E., Buccafusco J. J., Chapple C., de Groat W. C., Fryer A. D., Kay G., Laties A., Nathanson N. M., Pasricha P. J., Wein A. J. (2006). Muscarinic receptors: their distribution and function in body systems, and the implications for treating overactive bladder. Br. J. Pharmacol..

[cit2] Kruse A. C., Ring A. M., Manglik A., Hu J., Hu K., Eitel K., Hubner H., Pardon E., Valant C., Sexton P. M., Christopoulos A., Felder C. C., Gmeiner P., Steyaert J., Weis W. I., Garcia K. C., Wess J., Kobilka B. K. (2013). Activation and allosteric modulation of a muscarinic acetylcholine receptor. Nature.

[cit3] Strang C. E., Renna J. M., Amthor F. R., Keyser K. T. (2010). Muscarinic acetylcholine receptor localization and activation effects on ganglion response properties. Invest. Ophthalmol. Visual Sci..

[cit4] Prosser R. S. (2014). New pipelines for novel allosteric GPCR modulators. Biophys. J..

[cit5] Christopoulos A., Kenakin T. (2002). G protein-coupled receptor allosterism and complexing. Pharmacol. Rev..

[cit6] Wang L., Martin B., Brenneman R., Luttrell L. M., Maudsley S. (2009). Allosteric modulators of g protein-coupled receptors: future therapeutics for complex physiological disorders. J. Pharmacol. Exp. Ther..

[cit7] Mohr K., Trankle C., Holzgrabe U. (2003). Structure/activity relationships of M2 muscarinic allosteric modulators. Recept. Channels.

[cit8] Miao Y., Caliman A. D., McCammon J. A. (2015). Allosteric effects of sodium ion binding on activation of the m3 muscarinic g-protein-coupled receptor. Biophys. J..

[cit9] Haga K., Kruse A. C., Asada H., Yurugi-Kobayashi T., Shiroishi M., Zhang C., Weis W. I., Okada T., Kobilka B. K., Haga T., Kobayashi T. (2012). Structure of the human M2 muscarinic acetylcholine receptor bound to an antagonist. Nature.

[cit10] Vilardaga J. P., Bunemann M., Feinstein T. N., Lambert N., Nikolaev V. O., Engelhardt S., Lohse M. J., Hoffmann C. (2009). GPCR and G proteins: drug efficacy and activation in live cells. Mol. Endocrinol..

[cit11] Homer R. W., Swanson J., Jilek R. J., Hurst T., Clark R. D. (2008). SYBYL line notation (SLN): a single notation to represent chemical structures, queries, reactions, and virtual libraries. J. Chem. Inf. Model..

[cit12] Jawallapersand P., Mashele S. S., Kovacic L., Stojan J., Komel R., Pakala S. B., Krasevec N., Syed K. (2014). Cytochrome P450 monooxygenase CYP53 family in fungi: comparative structural and evolutionary analysis and its role as a common alternative anti-fungal drug target. PLoS One.

[cit13] Burger S. K., Lacasse M., Verstraelen T., Drewry J., Gunning P., Ayers P. W. (2012). Automated Parametrization of AMBER Force Field Terms from Vibrational Analysis with a Focus on Functionalizing Dinuclear Zinc(ii) Scaffolds. J. Chem. Theory Comput..

[cit14] Grubisic S., Brancato G., Pedone A., Barone V. (2012). Extension of the AMBER force field to cyclic alpha,alpha dialkylated peptides. Phys. Chem. Chem. Phys..

[cit15] Baron R., Vellore N. A. (2012). LSD1/CoREST is an allosteric nanoscale clamp regulated by H3-histone-tail
molecular recognition. Proc. Natl. Acad. Sci. U. S. A..

[cit16] Wang W., Ye W., Jiang C., Luo R., Chen H. F. (2014). New force field on modeling intrinsically disordered proteins. Chem. Biol. Drug Des..

[cit17] Huang Z., Zhu L., Cao Y., Wu G., Liu X., Chen Y., Wang Q., Shi T., Zhao Y., Wang Y., Li W., Li Y., Chen H., Chen G., Zhang J. (2011). ASD: a comprehensive database of allosteric proteins and modulators. Nucleic Acids Res..

[cit18] Garcia-Garcia C., Draper D. E. (2003). Electrostatic interactions in a peptide–RNA complex. J. Mol. Biol..

[cit19] Qin F., Chen Y., Wu M., Li Y., Zhang J., Chen H. F. (2010). Induced fit or conformational selection for RNA/U1A folding. RNA.

[cit20] Miller 3rd B. R., McGee Jr T. D., Swails J. M., Homeyer N., Gohlke H., Roitberg A. E. (2012). MMPBSA.py: An Efficient Program for End-State Free Energy Calculations. J. Chem. Theory Comput..

[cit21] Chen H. F. (2008). Mechanism of Coupled Folding and Binding in the siRNA-PAZ Complex. J. Chem. Theory Comput..

[cit22] Chen H. F., Luo R. (2007). Binding induced folding in p53-MDM2 complex. J. Am. Chem. Soc..

[cit23] Wang J., Cai Q., Xiang Y., Luo R. (2012). Reducing grid-dependence in finite-difference Poisson-Boltzmann calculations. J. Chem. Theory Comput..

[cit24] Wang J., Luo R. (2010). Assessment of linear finite-difference Poisson-Boltzmann solvers. J. Comput. Chem..

[cit25] Cai Q., Hsieh M. J., Wang J., Luo R. (2010). Performance of Nonlinear Finite-Difference Poisson-Boltzmann Solvers. J. Chem. Theory Comput..

[cit26] Sethi A., Eargle J., Black A. A., Luthey-Schulten Z. (2009). Dynamical networks in tRNA:protein complexes. Proc. Natl. Acad. Sci. U. S. A..

[cit27] Shannon P., Markiel A., Ozier O., Baliga N. S., Wang J. T., Ramage D., Amin N., Schwikowski B., Ideker T. (2003). Cytoscape: a software environment for integrated models of biomolecular interaction networks. Genome Res..

[cit28] Mirzarezaee M., Sadeghi M., Araabi B. N. (2011). Dynamical analysis of yeast protein interaction network during the sake brewing process. J. Microbiol..

[cit29] Girvan M., Newman M. E. (2002). Community structure in social and biological networks. Proc. Natl. Acad. Sci. U. S. A..

[cit30] Wang W., Jiang C., Zhang J., Ye W., Luo R., Chen H. F. (2016). Dynamics Correlation Network for Allosteric Switching of PreQ1 Riboswitch. Sci. Rep..

[cit31] Liu H., Ye W., Chen H. F. (2017). Positive cooperative regulation of double binding sites for human acetylcholinesterase. Chem. Biol. Drug Des..

[cit32] Guo X., Han J., Luo R., Chen H. F. (2017). Conformation Dynamics of the Intrinsically Disordered Protein c-Myb with the ff99IDPs Force Field. RSC Adv..

[cit33] Yang J., Liu H., Liu X., Gu C., Luo R., Chen H. F. (2016). Synergistic Allosteric Mechanism of Fructose-1,6-bisphosphate and Serine for Pyruvate Kinase
M2 *via* Dynamics Fluctuation Network Analysis. J. Chem. Inf. Model..

[cit34] Ye W., Qian T., Liu H., Luo R., Chen H. F. (2017). Allosteric Autoinhibition Pathway in Transcription Factor ERG: Dynamics Network and Mutant Experimental Evaluations. J. Chem. Inf. Model..

[cit35] Cai Y., Liu H., Chen H. (2018). Allosteric mechanism of quinoline inhibitors for HIV RT-associated RNase with MD simulation and dynamics fluctuation network. Chem. Biol. Drug Des..

[cit36] Zhang J. M., Jiang C., Ye W., Luo R., Chen H. F. (2016). Allosteric pathways in tetrahydrofolate sensing riboswitch with dynamics correlation network. Mol. BioSyst..

[cit37] Zhang J., Luo H., Liu H., Ye W., Luo R., Chen H.-F. (2016). Synergistic Modification Induced Specific Recognition between Histone and TRIM24 *via* Fluctuation Correlation Network Analysis. Sci. Rep..

[cit38] Rahman M. U., Liu H., Wadood A., Chen H. F. (2016). Allosteric mechanism of cyclopropylindolobenzazepine inhibitors for HCV NS5B RdRp *via* dynamic correlation network analysis. Mol. BioSyst..

[cit39] Silverbush D., Sharan R. (2014). Network orientation *via* shortest paths. Bioinformatics.

[cit40] del Sol A., Fujihashi H., Amoros D., Nussinov R. (2006). Residues crucial for maintaining short paths in network communication mediate signaling in proteins. Mol. Syst. Biol..

[cit41] Furini S., Barbini P., Domene C. (2013). DNA-recognition process described by MD simulations of the lactose repressor protein on a specific and a non-specific DNA sequence. Nucleic Acids Res..

[cit42] Gregory K. J., Hall N. E., Tobin A. B., Sexton P. M., Christopoulos A. (2010). Identification of orthosteric and allosteric site mutations in M2 muscarinic acetylcholine receptors that contribute to ligand-selective signaling bias. J. Biol. Chem..

[cit43] Kruse A. C., Hu J., Kobilka B. K., Wess J. (2014). Muscarinic acetylcholine receptor X-ray structures: potential implications for drug development. Curr. Opin. Pharmacol..

