# Supplementary material for: Synergistic regulation mechanism of iperoxo and LY2119620 for muscarinic acetylcholine M2 receptor
Source: RSC Adv. 2018 Apr 9;8(24):13067–74. doi: 10.1039/c8ra01545g (PMC9079678; doi:10.1039/c8ra01545g)
Supplement: RA-008-C8RA01545G-s001 [file RA-008-C8RA01545G-s001.pdf]

Electronic Supplementary Material (ESI) for RSC Advances.
This journal is © The Royal Society of Chemistry 2018

Supporting information

Synergistic Regulation Mechanism of Iperoxo and LY2119620 for Muscarinic Acetylcholine M2 Receptor

Quan Li[1] and Hai-Feng Chen[1,2,*]

[1]State Key Laboratory of Microbial metabolism, Department of Bioinformatics and Biostatistics, National Experimental Teaching Center for Life Sciences and Biotechnology, College of Life Sciences and Biotechnology, Shanghai Jiaotong University, Shanghai, 200240, China
[2]Shanghai Center for Bioinformation Technology, Shanghai, 200235, China

*Corresponding authors
Email addresses: haifengchen@sjtu.edu.cn
Tel: 86-21-34204348
Fax: 86-21-34204348.

The authors declare that there is no conflict of interest.

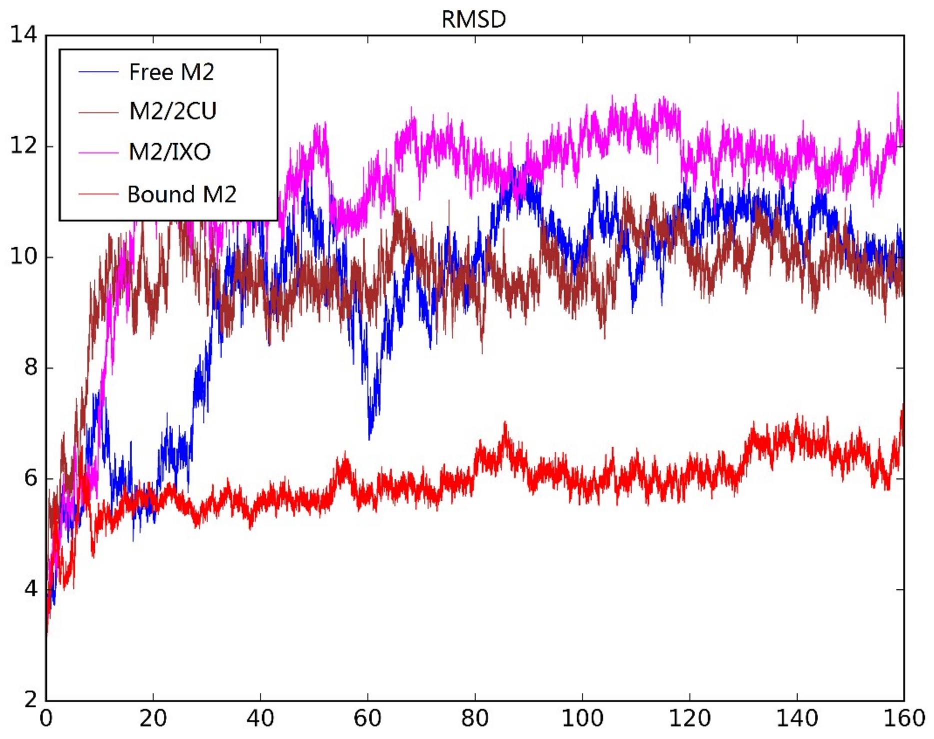

Figure S1. Root mean squared fluctuations of Cα atom for four systems.

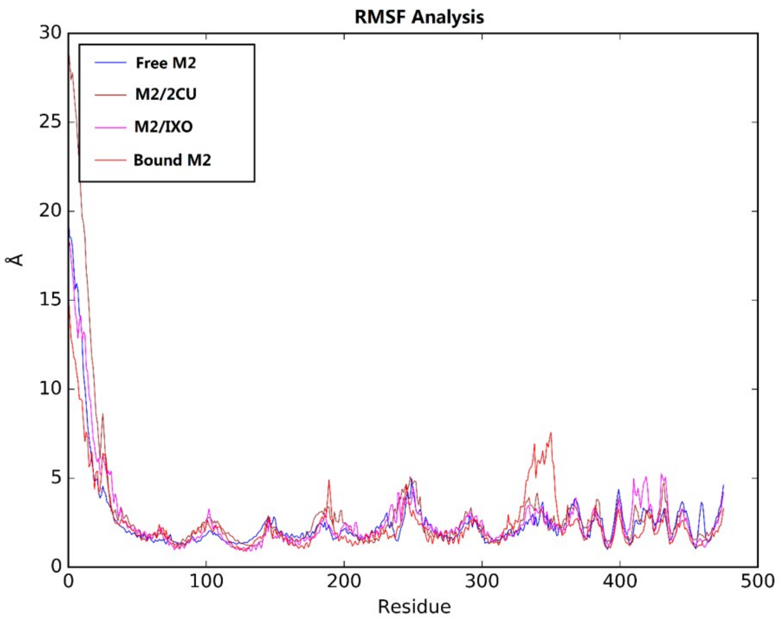

Figure S2. Average fluctuations of Cα atoms for four systems.

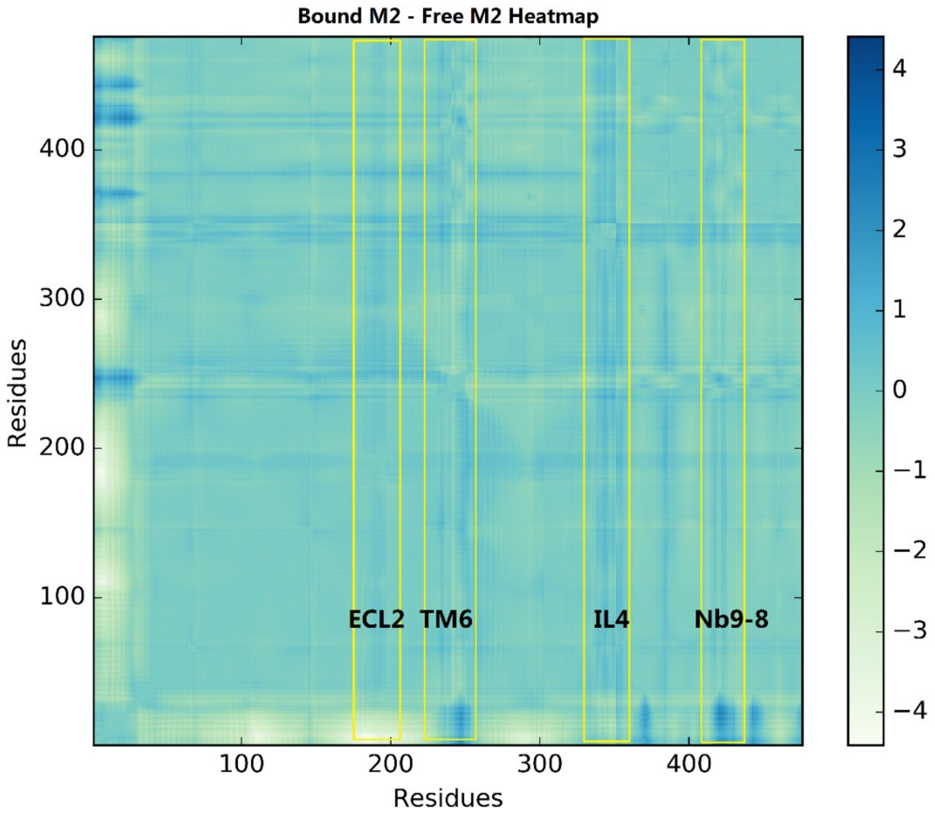

Figure S3. Distance different landscape between bound and free M2.

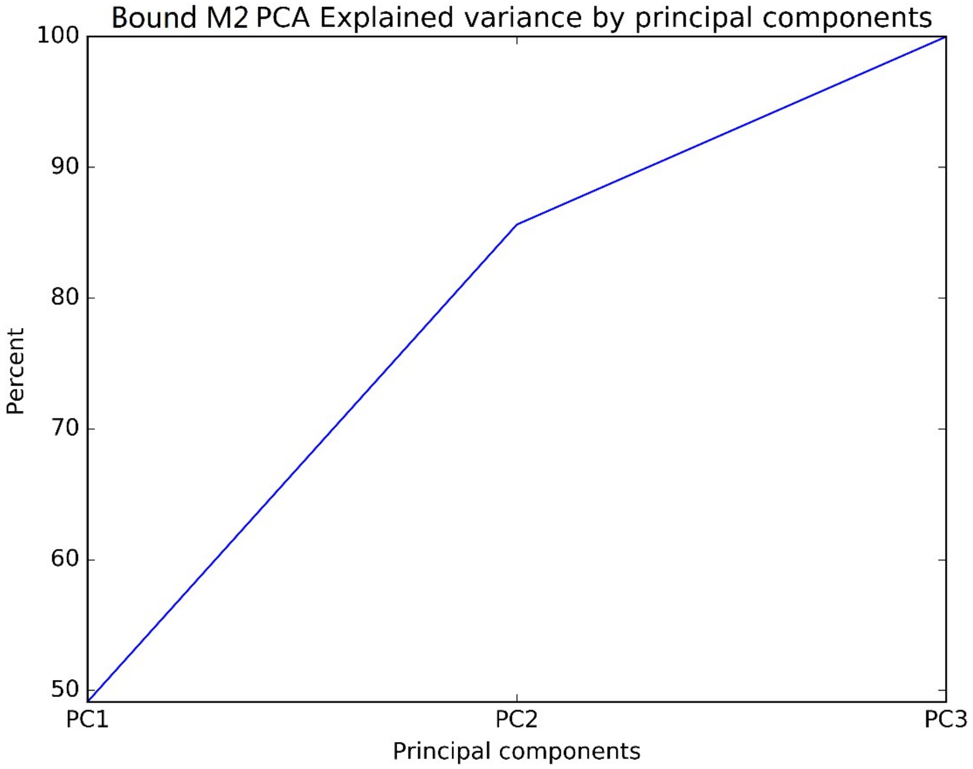

Figure S4. PCA Explained variance by principal components

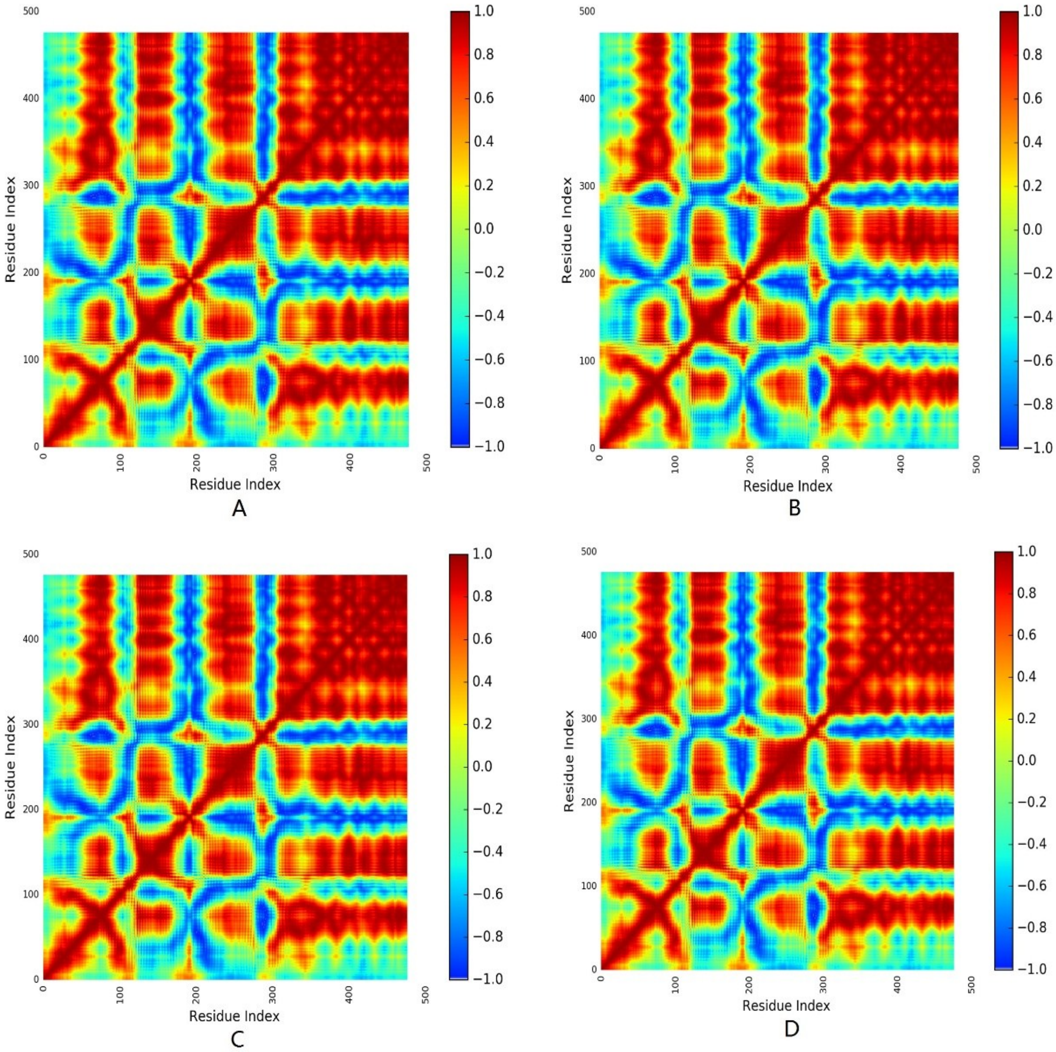

Figure S5. Dynamic Cross-Correlation Maps for each system. A: Free M2. B: M2/IXO. C: M2/2CU. D: Bound M2.

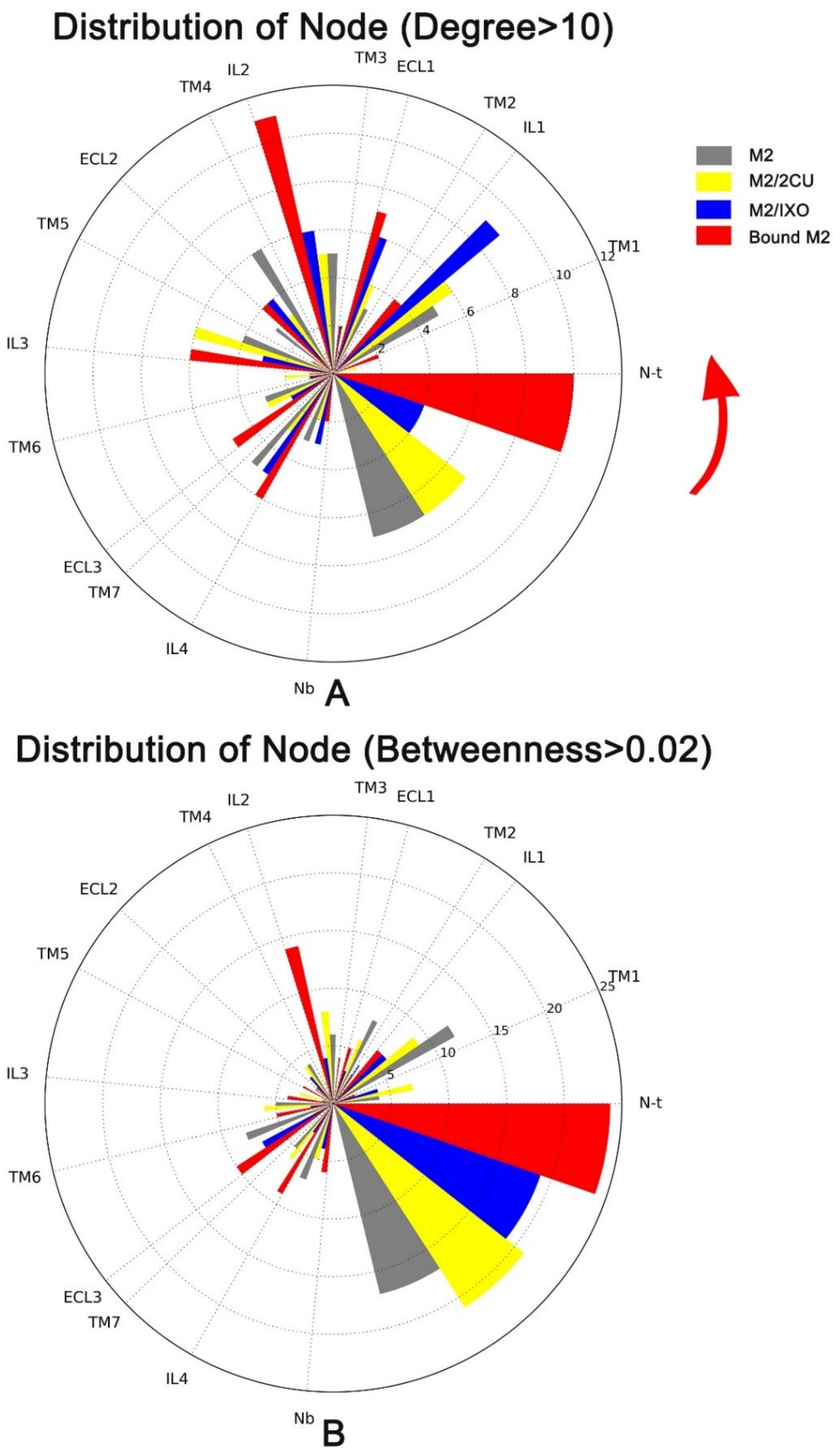

Figure S6. The distribution of nodes with weighted degree (>10) and weighted betweenness (>0.02). A: Distribution of node (degree>10). B: Distribution of node (betweenness >0.02).

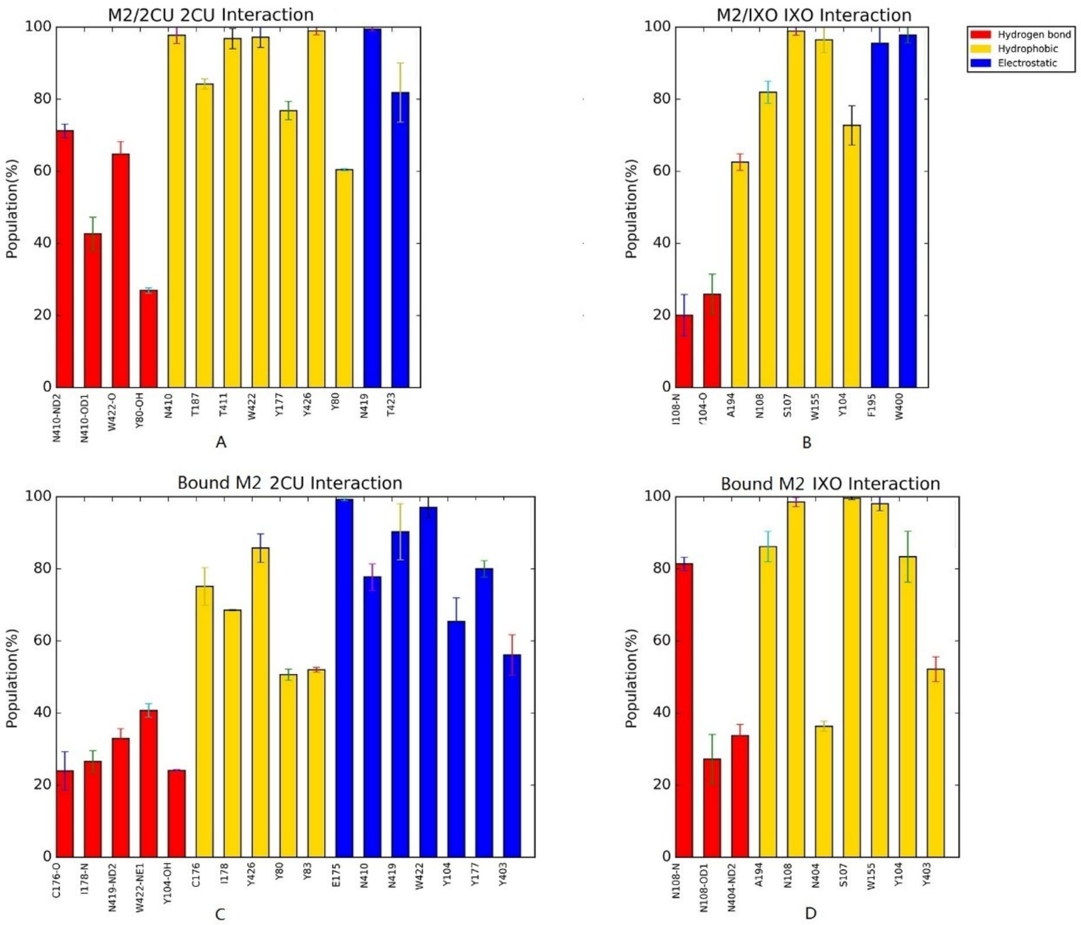

Figure S7. Interaction between M2 and ligand for four system. A: Interaction between 2CU and M2 for M2/2CU. B: Interaction between IXO and M2 for M2/IXO. C: Interaction between 2CU and M2 for Bound M2. D: Interaction between IXO and M2 for Bound M2.

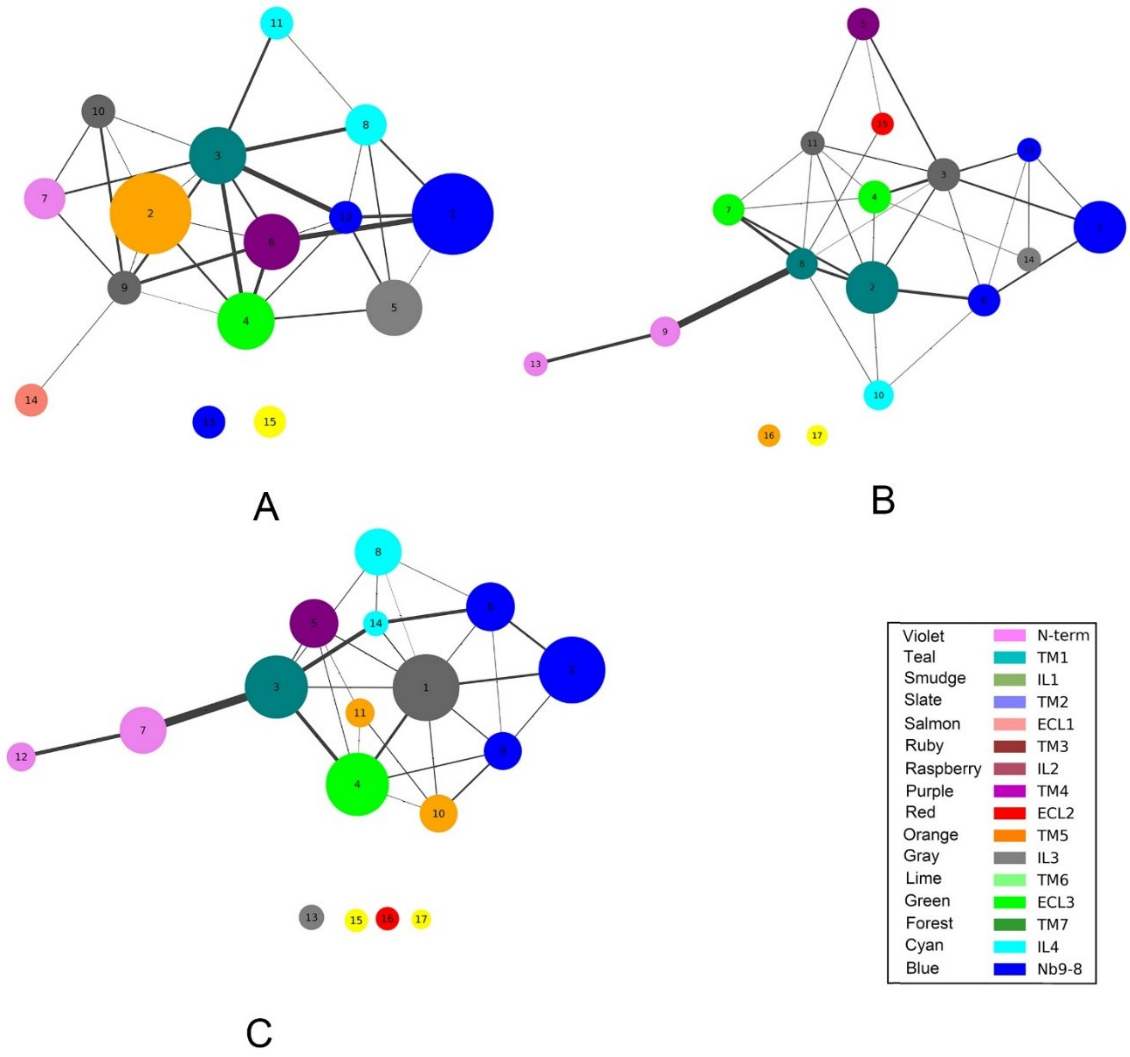

Figure S8. Modifications to perturb the community network for Bound M2. A: IXO-weakened. B: 2CU-weakened. C: IXO&2CU-weakened.

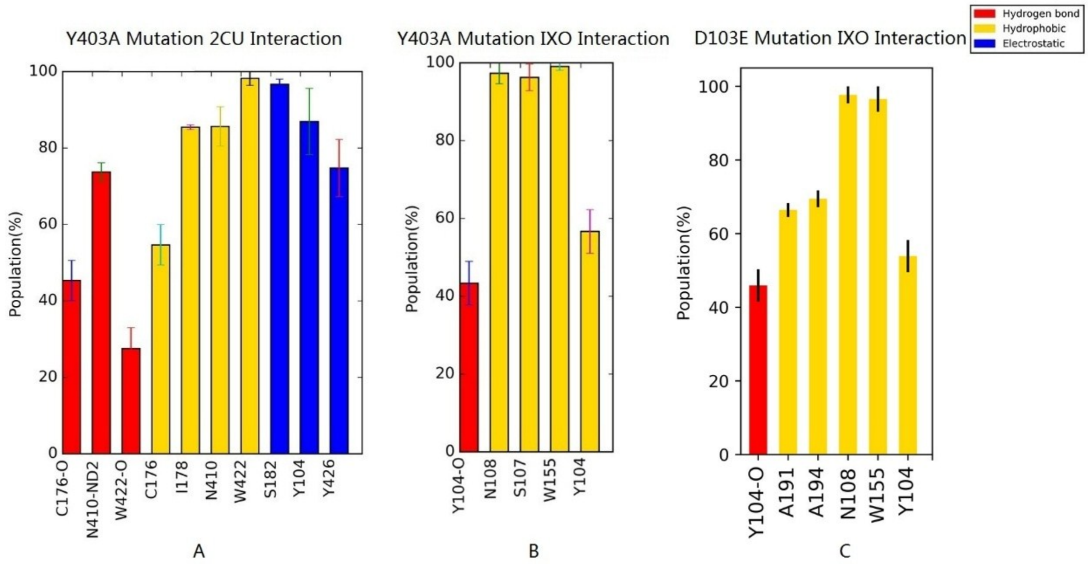

Figure S9. Interaction between M2 and ligand for Y403A and D103E mutant. A: Interaction between 2CU and M2 for Bound M2. B: Interaction between IXO and M2 for Bound M2. C: Interaction between IXO and M2 for D103E.

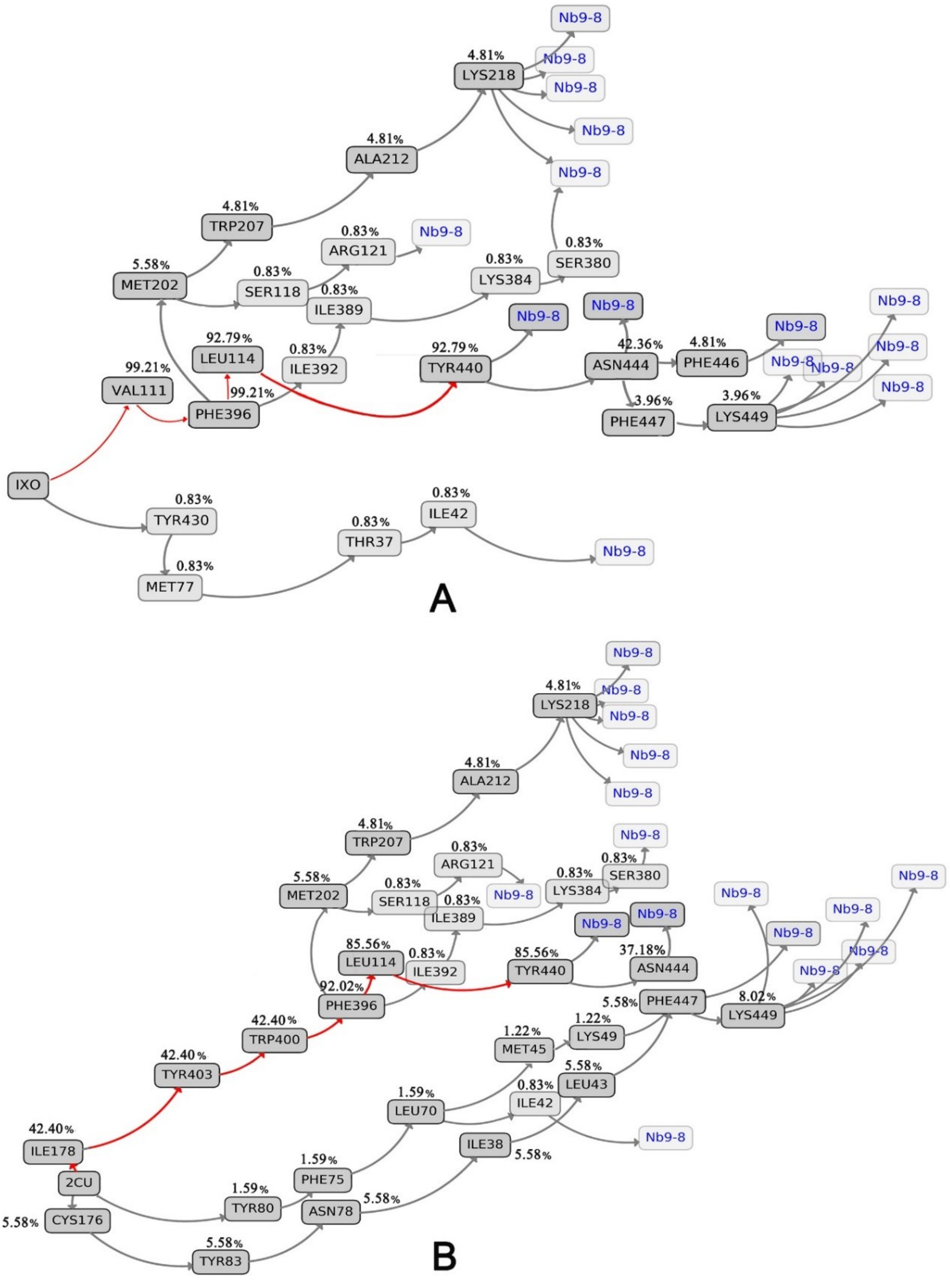

Figure S10. Allosteric regulation pathways of bound M2. A: IXO regulation pathway. B: 2CU regulation pathway.

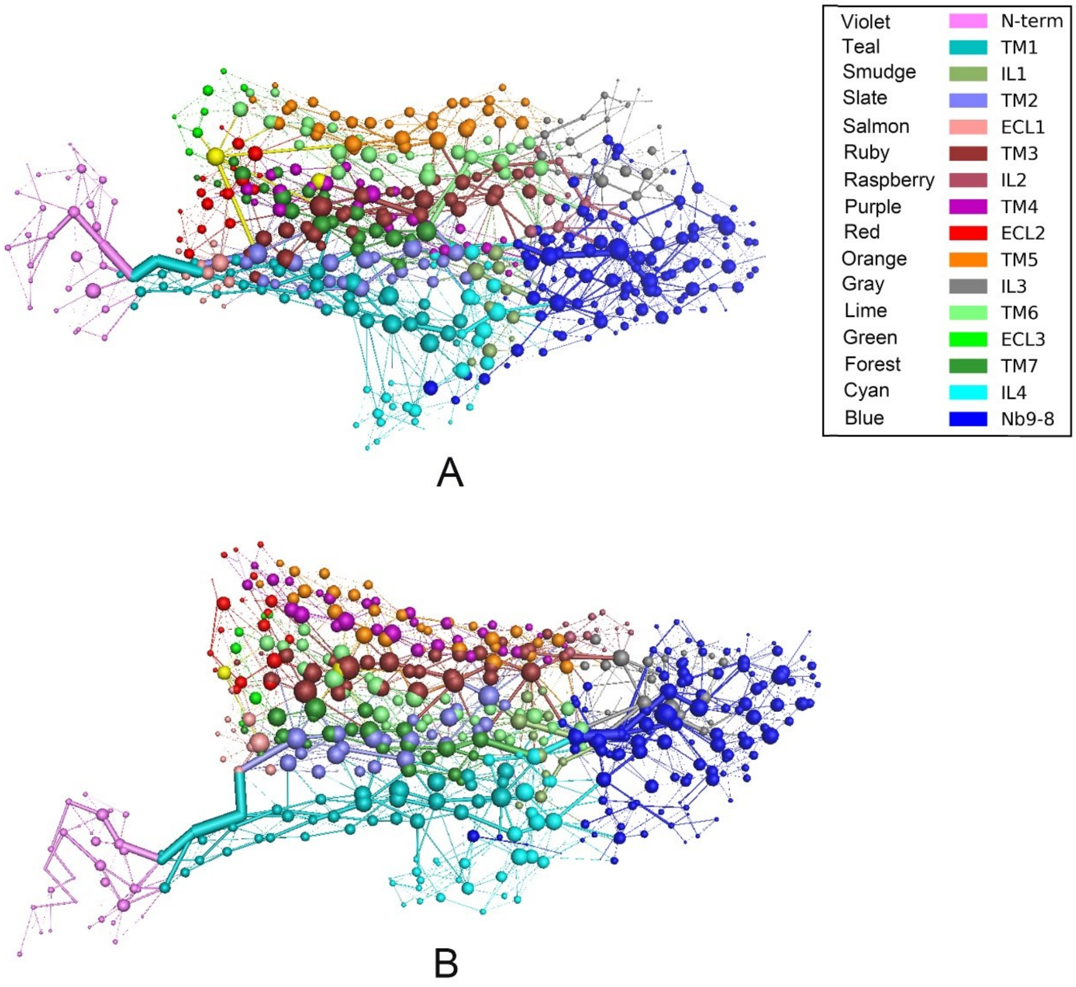

Figure S11. Dynamics network for Y403A and F396A mutant. A: Network for Y403A mutant. B: Network for F396A mutant.

.

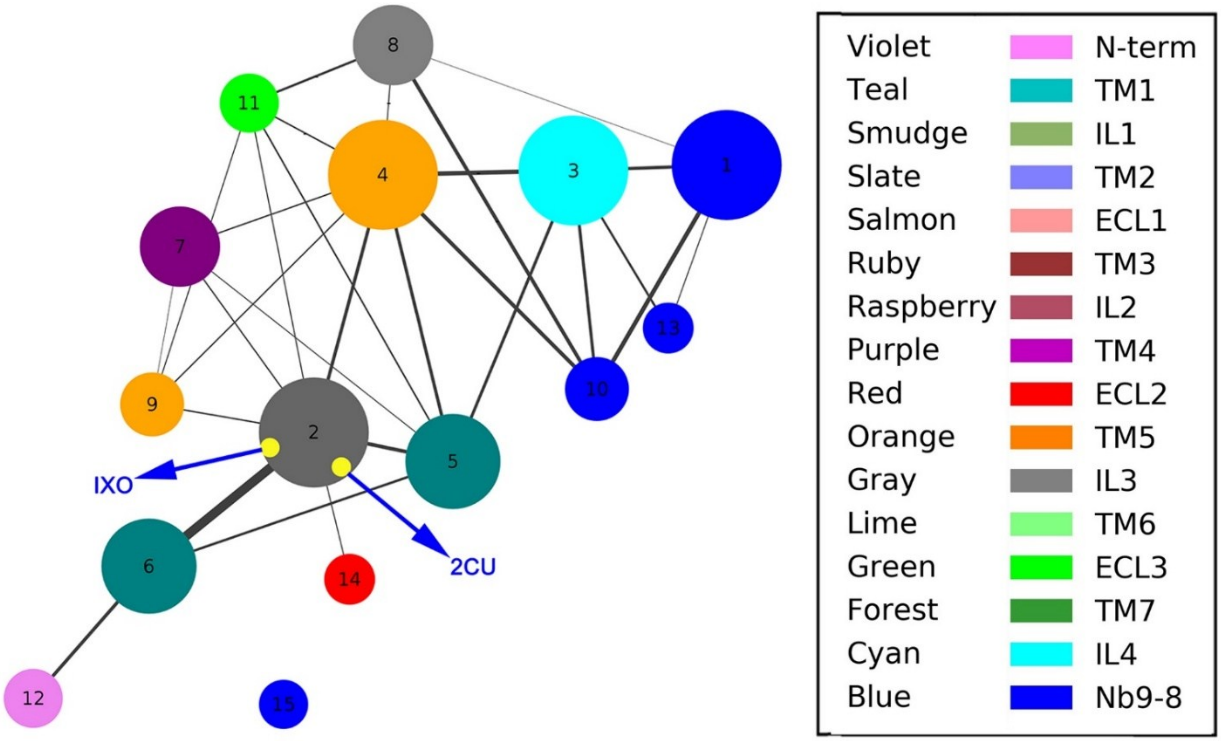

Figure S12. Community Network for F396A mutant.

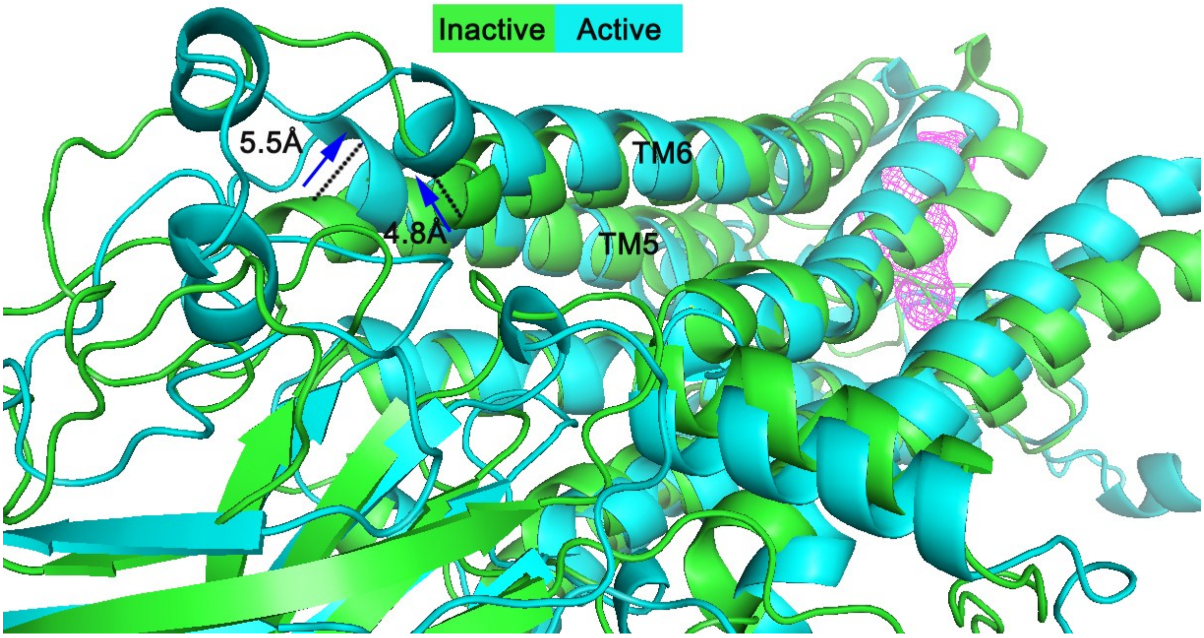

Figure S13. Alignment between inactive and active states. TM5 and TM6 are closer in M2-Active crystal structures of GPCRs reveal a network of hydrogen bonding interactions that extend from the binding pocket to the cytoplasmic surface.

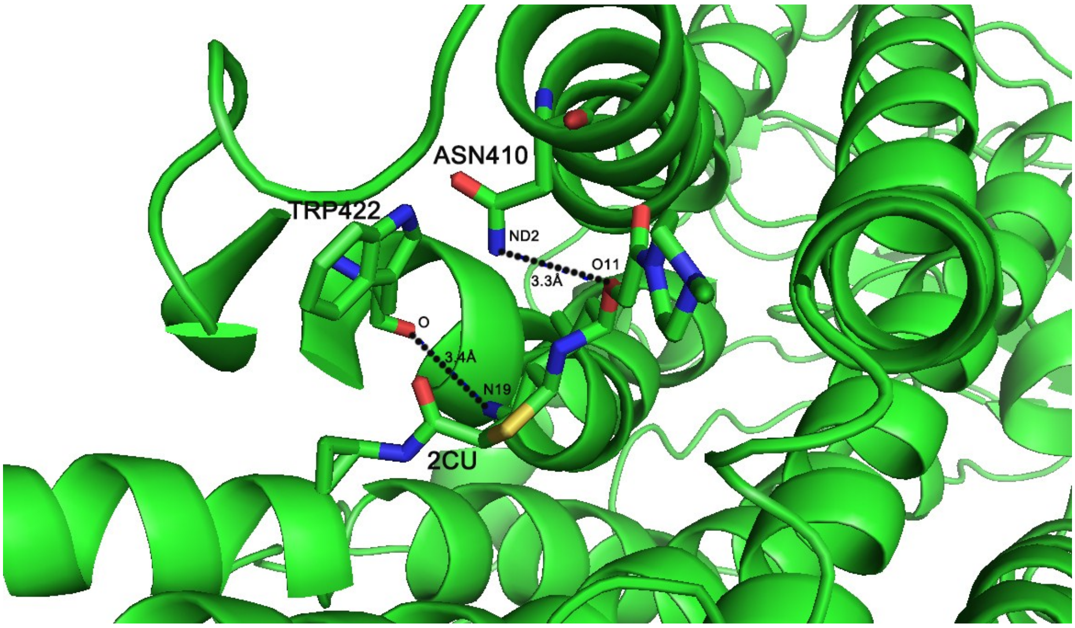

Figure S14. Two hydrogen bonds with TRP422 and ASN410.
